# Towards a revision of the genus *Periclimenes*: resurrection of *Ancylocaris* Schenkel, 1902, and designation of three new genera (Crustacea, Decapoda, Palaemonidae)

**DOI:** 10.3897/zookeys.646.11397

**Published:** 2017-01-17

**Authors:** Zdeněk Ďuriš, Ivona Horká

**Affiliations:** 1 Department of Biology and Ecology and Institute of Environmental Technologies, Faculty of Science, University of Ostrava, Chittussiho 10, CZ-710 00 Ostrava, Czech Republic

**Keywords:** Ancylocaris, Actinimenes, Cristimenes, Periclimenes, Rapimenes, symbiotic shrimps

## Abstract

Based on recently published molecular phylogenies of Indo-West Pacific palaemonid shrimps and further morphological evidence, the systematic position of several species of the polyphyletic genus *Periclimenes* is revised. The generic name *Ancylocaris* Schenkel, 1902 is re-established for the anemone-associated *Periclimenes brevicarpalis*. *Actinimenes* **gen. n.**, is proposed for the anemone-associated *Periclimenes inornatus*, *Periclimenes ornatellus* and *Periclimenes ornatus*, all of which have a subspatulate first pereiopod. *Cristimenes* **gen. n.**, is designated for the echinoderm-associated species, *Periclimenes commensalis*, *Periclimenes cristimanus*, and *Periclimenes zanzibaricus*, all with a unique carpo-propodal articulation of the second pereiopods. *Rapimenes* **gen. n.** is established for the hydroid and antipatharian-associated *Periclimenes brucei*, *Periclimenes granulimanus*, and *Periclimenes laevimanus*, for which the long, slender and unequal second pereiopods and prehensile ambulatory propodi are the main synapomorphic characters.

## Introduction

The apparently polyphyletic and highly diverse palaemonid shrimp genus *Periclimenes* O. G. Costa, 1844 (e.g. Bruce et al. 2005) has in recent decades undergone some splitting by the designation of new genera for several species groups, e.g. *Exoclimenella* Bruce, 1995, *Periclimenella* Bruce, 1995, *Manipontonia* Bruce, Okuno & Li, 2005, *Crinotonia* Marin, 2006, *Brucecaris* Marin & Chan, 2006, *Unguicaris* Marin & Chan, 2006, *Margitonia* Bruce, 2007, *Leptomenaeus* Bruce, 2007, *Rapipontonia* Marin, 2009, and *Ancylomenes* Okuno & Bruce, 2010. Equally, several synonymised genera were re-established, e.g. *Harpilius* Dana, 1852, *Urocaris* Stimpson, 1860, *Laomenes* AH Clark, 1919 and *Cuapetes* AH Clark, 1919 (see Bruce 2004, 2007a, b, c, Okuno and Fujita 2007, Okuno 2009). However, the genus *Periclimenes* as presently delineated still represents a heterogeneous assemblage of taxa of unresolved systematic status.

Although several molecular studies were recently constructed (Kou et al. 2013, Gan et al. 2015, Horká et al. 2016) to examine intrageneric relationships of the genus *Periclimenes*, a full resolution has not been achieved yet, partly due to incomplete coverage of species diversity, partly due to the low basal support of some clades within the analysis. Excluding the species recently allocated to *Echinopericlimenes* Marin & Chan, 2014 and *Bathymenes* Kou, Li & Bruce, 2016, only about 25 species of *Periclimenes* are involved in the analyses representing about 20% from the almost 130 species presently in the genus.

*Periclimenes* is clearly a genus which will see a further, strong reduction of its species diversity in the future. The type species, *Periclimenes amethysteus* (Risso, 1827) is a member of a clade of four sea anemone associated species distributed in the Mediterranean Sea and neighbouring part of the eastern Atlantic Ocean. It seems quite probable that only those species, with perhaps a few allied Atlantic species, will remain in *Periclimenes*, while all other species are likely to require allocation to further new or indeed resurrected genera. As indicated from the phylogenetic reconstruction provided by Horká et al. (2016), this group of anemone shrimps may be related to the majority of other Atlantic *Periclimenes* species, including the western Atlantic anemone shrimps, as well as Atlantic and Indo-Pacific deep-water *Periclimenes*, *Echinopericlimenes*, *Altopontonia* Bruce, 1990, and *Bathymenes* species. As the systematic relationship of these taxa still remains poorly supported in the recent phylogenies, and due to the lack of inclusion of many other Atlantic taxa, the exact composition of *Periclimenes* (*s.s.*) thus remains unclear.

Nevertheless, it is evident from the phylogeny in Horká et al. (2016) that most Indo-West Pacific representatives of the genus (as currently defined) are unrelated to the Atlantic *Periclimenes* taxa as a whole, and some natural groups can easily be separated from the genus based on molecular and morphological support. This report is a contribution to a series of revisions of the genus, resurrecting a synonym and establishing three new genera for some species of Indo-West Pacific *Periclimenes* species.

Abbreviations: fcn, field collection number; MHNG, Muséum d’Histoire Naturelle, Geneve; MNHN, Muséum National d’Histoire Naturelle, Paris; MTQ, Museum of Tropical Queensland, Townsville; RMNH, Naturalis Biodiversity Centre (formerly Rijksmuseum van Natuurlijke Historie), Leiden; spm(s), specimen(s); sp./spp., species (single/plural); UO, University of Ostrava.

## Systematics

### Superfamily Palaemonoidea Family Palaemonidae Rafinesque, 1815

#### Genus Ancylocaris

Taxon classificationAnimaliaDecapodaPalaemonidae

Schenkel, 1902

##### Type species.

*Ancylocaris brevicarpalis* Schenkel, 1902, by monotypy.

##### Included species.

**Figure 1.**
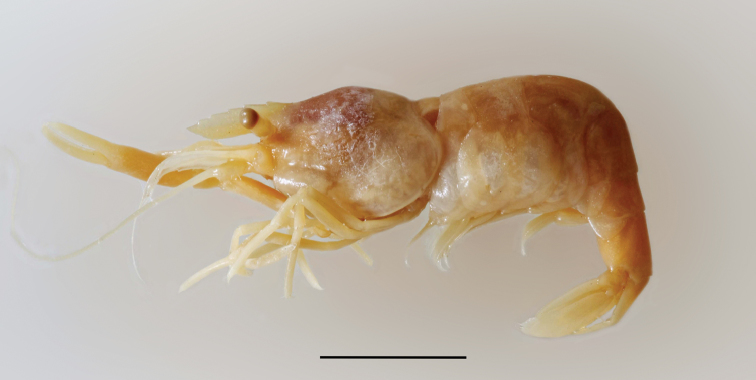
Holotype of *Palaemonella amboinensis* Zehntner, 1894, adult female, MHNG (photo: L Monod; scale bar 5 mm).

*Ancylocaris brevicarpalis* Schenkel, 1902 (Figs 1, 3A, B).

##### Gender.

Feminine.

##### Diagnosis.

Subcylindrical body form. Carapace smooth; rostrum well developed, subequal to antennular peduncle and moderately high, dorsal margin convex, dentate, with first tooth postorbital, ventral margin convex, with 1–2 teeth on distal third of rostrum length. Inferior orbital angle produced, without reflected inner flange, supraorbital and epigastric teeth absent, antennal and hepatic teeth present. Fourth thoracic sternite with broad transverse ridge subdivided by deep narrow median incision. Pleon smooth, third tergite non-carinated or posteriorly produced, pleura 1–5 posteroventrally rounded; telson with 2 pairs of minute dorsal spines on distal third of telson length and 3 pairs of short posterior marginal spines. Ophthalmic somite without interocular process. Antennule and antenna as usual for the family; upper ramus of antennular flagellum biramous, with fused basal part; scaphocerite moderately broad, with small distolateral tooth falling short of anterior margin of lamina; carpocerite short. Eyes with small accessory pigment spot dorsally on corneal margin. Mandible without palp; molar and incisor processes normal. Maxilla with basal endite distinctly bilobed, coxal endite obsolete, scaphognathite normal; first maxilliped with endites fused, exopod well developed, with multiple terminal setae, caridean lobe normal, epipod feebly bilobed; second maxilliped with normal endopod, exopod as in first maxilliped, without caridean lobe, epipod small, simple, without podobranch; third maxilliped with slender endopod, ischiomerus fused or feebly separated from basis, exopod as in second maxilliped, coxa with semi-circular lateral plate, single arthrobranch present. First pereiopods slender, coxa with distomedial setose lobe, fingers of chelae elongate, with lateral cutting edges. Second pereiopods moderately stout, similar and subequal, chelae with fingers kept laterally; fingers subequal to palm, cutting edges with simple lamina and 2 low proximal teeth, carpo-propodal articulation simple, carpus much shorter than palm in adults, feebly cup-shaped. Ambulatory pereiopods slender, propodus without ventral spines, dactyli with minute or reduced distoventral tooth on stout corpus, unguis elongate, curved. Endopod of male first pleopod simple, elliptic, with multiple spinules medioproximally and multiple pappose setae distally. Male second pleopod with appendix masculina slender, with several simple terminal and lateral setae. Uropods normal.

##### Figures

**(selected).** Schenkel (1902: Pl. 13, fig. 21), Kemp (1922: figs 40–42, Pl. 6, fig. 8), Kubo (1940: figs 13–14), Miyake and Fujino (1968: fig. 4), Bruce (1978: fig. 6; 1979: Pl. 1, fig. A), Fransen (1989: fig. 1a–c).

##### Systematic position.

*Ancylocaris brevicarpalis* (under the name *Periclimenes brevicarpalis*), together with *Periclimenes inornatus* Kemp, 1922, *Periclimenes nevillei* Bruce, 2010, *Periclimenes ornatus* Bruce, 1969, *Periclimenes ornatellus* Bruce, 1979, and *Periclimenes albolineatus* Bruce & Coombes, 1997, were previously believed to be members of a “*Periclimenes brevicarpalis*” group (see Bruce and Svoboda 1983, Bruce and Coombes 1997, Bruce 2010) some of which are sea anemone associated species. On the contrary, Fransen (1989) stated that only three of those species, i.e. *Periclimenes inornatus*, *Periclimenes ornatellus*, and *Periclimenes ornatus*, are closely related to each other and comprise a “*Periclimenes inornatus*” group (see *Actinimenes* gen. n., below), rather than belong in the “*Periclimenes brevicarpalis*” group. The available comprehensive molecular phylogenies (Gan et al. 2015, Horká et al. 2016) show that at least *Ancylocaris brevicarpalis* (under the name *Periclimenes brevicarpalis*) occupies a position away from the *Periclimenes inornatus* group.

While the species of the “*Periclimenes inornatus*” group share with *Ancylocaris* the general shape of the body, especially of the rostrum and the second pereiopods, they may easily be distinguished from *Ancylocaris brevicarpalis* by the presence of deeply subspatulate chelae of the first pereiopod, but also by the more numerous proximal teeth on the fingers of the second pereiopod, as well as larger and more anteriorly placed dorsal telson spines (the first pair before mid-length). The propodal segment of the second maxilliped in *Ancylocaris brevicarpalis* is broader than the dactylus and distomesially expanded, while sub-equally broad in the “*Periclimenes inornatus*” group (e.g. Kubo 1940, Bruce 1979).

The sister taxon for *Ancylocaris*, as revealed by the analyses of Gan et al. (2015) and Horká et al. (2016), is actually a pair of *Periclimenes* species, the crinoid-associated *Periclimenes affinis* (Zehntner, 1894) and *Periclimenes kallisto* Bruce, 2008, which is symbiotic with antipatharian corals. The significant genetic distance of this pair however indicates that their position is most likely quite distant from *Ancylocaris*. Both those species show some resemblance to *Ancylocaris brevicarpalis* in the size and position of the dorsal telson dentition, and in the distomedial coxal lobe and fingers on the first pereiopods. *Periclimenes affinis* also has a short carpocerite and a similar carpus of the second pereiopod, and similar male pleopod shape and setation. *Periclimenes kallisto* has feeble dentition of the fingers of the second pereiopod and the ambulatory dactyli with a minute distoventral tooth. These two species are more slender and smaller than *Ancylocaris brevicarpalis* and bear a slender rostrum with obsolete ventral carina, unequal second pereiopods, ventrally spinulose ambulatory propodi, and the endopod of the male first pleopod has a distomedial lobe (Holthuis 1958, Bruce 1980, 2008a). A close affinity between these two species was suggested by Bruce (2008a), who highlighted a group of species of a similar morphology, additionally including *Periclimenes canalinsulae* Bruce & Coombes, 1997, and *Periclimenes jugalis* Holthuis, 1952. Some other taxa, for instance *Periclimenes novaffinis* Bruce & Coombes, 1997, *Periclimenes albolineatus* and *Periclimenes nevillei*, may also belong to this group. The systematic relation of this assemblage as well as of each particular member to the genus *Ancylocaris* remains to be resolved.

##### Remarks.

The earliest report on the present species was published by Zehntner in 1984. However, Holthuis (1952) places *Palaemonella amboinensis* Zehntner, 1894 into the synonymy of *Ancylocaris brevicarpalis* Schenkel, 1902 under the name *Periclimenes brevicarpalis* (Schenkel, 1902), as he considers the drawing of the scaphocerite and the antennular peduncle in Zehntner (1894) not to be entirely correctly drawn. After the examination of a photograph of the holotype (Fig. 1) kindly provided by L. Monod (MHNG) we fully concur with this position. *Palaemonella amboinensis* Zehntner (1894) should thus have priority over *Periclimenes brevicarpalis* (Schenkel, 1902); however as stated by Holthuis (1952) the latter name is preoccupied by *Periclimenes amboinensis* (De Man, 1888), originally described as *Anchistia amboinensis* De Man, 1888. For some time now both taxa are no longer considered congeneric, as *Anchistia amboinensis* De Man, 1888 was placed in the genus *Laomenes* AH Clark, 1919, resurrected for a group of crinoid dwelling species by Okuno and Fujita (2007). Conversely, *Ancylocaris brevicarpalis* Schenkel, 1902 was maintained in the genus *Periclimenes* up to now, although now returned to the resurrected genus *Ancylocaris*. This creates some ambiguity as to what is the correct name for the taxon currently known as *Periclimenes brevicarpalis* (Schenkel), a rather widespread, well-known and often photographed species.

Article 60.1 (ICZN 1999) specifies that a junior homonym must be rejected and replaced either by an available and potentially valid synonym or, for lack of such a name, by a new substitute name. We herein interpret Holthuis’s (1952) action in proposing to use a junior synonym, *Ancylocaris brevicarpalis* Schenkel, 1902, for *Palaemonella amboinensis* Zehntner, 1894 as a “substitute name”. In which case, Art. 59.3 specifies “that a junior secondary homonym replaced before 1961 is permanently invalid unless the substitute name is not in use and the relevant taxa are no longer considered congeneric, in which case the junior homonym is not to be rejected on grounds of that replacement”. Clearly, the substitute name, *Periclimenes brevicarpalis* (Schenkel) is in widespread use, throughout the scientific literature as well as popular accounts, as it is one of the most photographed shrimp species. Even though both taxa have not been considered congeneric since the resurrection of the genus *Ancylocaris* makes *Ancylocaris brevicarpalis* Schenkel, 1902 the valid name for the species in question.

##### Distribution.

The single species in the genus is widely distributed throughout the whole Indo-West Paciﬁc, from South Africa and Red Sea to Japan and Polynesia.

##### Ecology.

*Ancylocaris brevicarpalis* is obligatory associated with sea anemones (Cnidaria: Actiniaria) (cf. Fransen 1989, Müller 1993), although juveniles may also occur on alcyonarian and scleractinian corals.

#### Actinimenes gen. n.

Taxon classificationAnimaliaDecapodaPalaemonidae

http://zoobank.org/A1D1A9D6-406C-4EB3-B750-494A81EEAF9A

##### Type species.

*Periclimenes ornatus* Bruce, 1969, by present designation.

##### Included species.

*Actinimenes inornatus* (Kemp, 1922), comb. n. (Fig. 3C); *Actinimenes ornatus* (Bruce, 1969), comb. n. (Fig. 3D); and *Actinimenes ornatellus* (Bruce, 1979), comb. n.

##### Diagnosis.

Carapace smooth; rostrum well developed, compressed, dorsal and ventral margins convex, with 7–10 dorsal teeth (posterior tooth behind orbits) and 0–2 ventral teeth, lateral carinae and orbit feebly developed, epigastric and supraorbital spines absent, inferior orbital angle usually produced, rounded, antennal tooth marginal, hepatic tooth close to level of latter. Pleon smooth, third segment not posteriorly produced, pleura rounded. Telson with two pairs of moderately large dorsal marginal spines situated on anterior and posterior thirds of telson length; three pairs of posterior spines, lateral spines smaller than dorsal spines. Eyes with globular cornea, small additional pigment spot dorsally on corneal margin. Antennule well developed. Antennal basicerite armed with lateral tooth; scaphocerite well developed, moderately broad, with distolateral tooth small, not reaching distal end of lamella. Mandible without palp, molar process robust, incisor process as usual for the family. Maxillula with bilobed palp, laciniae as usual for the family. Maxilla with simple palp, basal endite slender, deeply bilobed, coxal endite obsolete, scaphognathite moderately broad. First maxilliped with simple palp, basal endite fused with coxal endite, exopod with large caridean lobe, ﬂagellum slender with several plumose distal setae, epipod feebly bilobed. Second maxilliped with normal endopod, propodus not produced distomesially, exopod similar to ﬁrst maxilliped, without accessory lobe, coxa with oval epipod without podobranch. Third maxilliped with slender endopod, ischiomerus fused to basis, exopod as in second maxilliped, coxa with oval lateral plate, arthrobranch rudimentary or lacking. Fourth thoracic sternite with broad transverse ridge subdivided by median incision. First pereiopods moderately slender, chela with ﬁngers subequal to palm, deeply subspatulate with entire cutting edges, coxa with setose distoventral lobe. Second pereiopods well developed, smooth, similar and equal, ﬁngers with several small recurved teeth on proximal half, palm subcylindrical, longer than fingers, carpo-propodal articulation terminal, carpus much shorter than palm, merus unarmed, coxa without distoventral lobe. Ambulatory pereiopods moderately slender, propodus without ventral spines, dactyli with stout unarmed corpus, unguis elongate, curved. Endopod of male first pleopod simple, elliptic, feebly spinulose medioproximally, with several setulose setae distolaterally. Male second pleopod with appendix masculina slender, with several simple terminal and lateral setae. Uropods normal, exopod with small distolateral tooth and normal movable spine.

##### Etymology.

From Actiniaria, the order of Anthozoa which comprises the host sea anemones for the genus, and *Periclimenes* to which genus the species previously belonged; gender masculine.

##### Figures

**(selected).** Kemp (1922: figs 43–46), Bruce (1976: 10–11; 1979: figs 3B, 4–7, Pl. 1: fig. B–E; 1982: figs 11–12), Fransen (1989: figs 2–3).

##### Systematic position.

Based on recent molecular studies (Gan et al. 2015, Horká et al. 2016), species of the genus *Actinimenes* gen. n. show a close phylogenetic relationship to two groups of taxa, *Zenopontonia* Bruce, 1975 and some other echinoderm-associated taxa on the one hand, and to the *Periclimenes diversipes* species group on the other hand. While the latter are also cnidarian associates, they are distinctly smaller species with more slender ambulatory pereiopods and dactyls, but mainly with very distinctive second pereiopods with both or at least one of the chelae of a specific subspatulate shape, with fingers generally longer than palm. Further, the species of the latter group share a similar shape of rostrum, and position of the carapacial teeth, subspatulate first pereiopod chela, short carpus of second pereiopods, simple ambulatory dactyli with elongate sharp unguis, and the shape and spinulation of the male pleopods (Bruce 1989).

*Zenopontonia* as well as other related echinoderm-associated taxa, such as *Periclimenes colemani* Bruce, 1975, and *Lipkemenes lanipes* (Kemp, 1922), are generally also very similar to *Actinimenes* by the position of antennal and hepatic teeth, an incised transverse ridge on the fourth thoracic sternite, the deeply subspatulate chelae of the first pereiopod, the shape of the chela of the second pereiopod and the very short carpus, and by the shape and spinulation of the male pleopods (Bruce 1989). These species however differ from *Actinimenes* spp. by a more down curved rostrum, more posteriorly situated and smaller dorsal telson spines, fine pectination on the fingers of the first pereiopod, and the ambulatory dactyli having small distoventral tooth on the corpus, sometimes also fully reduced (Marin 2012). Some mollusc- or ascidian-associated genera, e.g. *Anchistus* Borradaile, 1898, *Paranchistus* Holthuis, 1952, or *Dasella* Lebour, 1945, show some phylogenetic relationship to these echinoderm associated taxa (Gan et al. 2015, Horká et al. 2016), and thus more remotely also to *Actinimenes* gen. n. All species of these genera differ however from *Actinimenes* gen. n. by specialized biunguiculate ambulatory dactyli.

The three species of *Actinimenes* gen. n. were previously thought to be part of the ‘*Periclimenes brevicarpalis* group’ (Bruce and Svoboda 1983, Bruce 2010), although Fransen (1989) regarded them as comprising a ‘*Periclimenes inornatus* group’ of identical composition of the new genus (see also Remarks for *Ancylocaris brevicarpalis* comb. n., above).

##### Distribution.

Widely distributed in the Indo-West Paciﬁc from the Red Sea and Kenya to Japan, Marshall Islands, and Fiji.

##### Ecology.

The species of the present genus are all obligate associates of sea anemones (Cnidaria: Actiniaria) (see Fransen 1989, Müller 1993).

##### Key to species identification of *Actinimenes* gen. n.

**Table:** 

1	Fourth thoracic sternite produced anteriorly, bilobed, lobes abutting but separated by narrow deep incision; colouration: body with dense longitudinal lines of orange dots, pereiopods dark purple/red spotted	***Actinimenes ornatus* (Bruce, 1969), comb. n.**
–	Fourth thoracic sternite with U-shaped median incision; colouration: generally transparent except white band between eyes; adult females with white median band on bottom of thoracic and abdominal segments	**2**
2	Ambulatory dactyli with unguis proximo-ventrally smooth (adult females with white median band on thoracic and abdominal segments)	***Actinimenes inornatus* (Kemp, 1922), comb. n.**
–	Ambulatory dactyli with unguis proximo-ventrally serrated	***Actinimenes ornatellus* (Bruce, 1979), comb. n.**

#### Cristimenes gen. n.

Taxon classificationAnimaliaDecapodaPalaemonidae

http://zoobank.org/4A7068CC-CB18-4D6B-A9B0-FD518F4B3F19

##### Type species.

Periclimenes (Cristiger) commensalis Borradaile, 1915, by present designation.

##### Included species.

**Figure 2.**
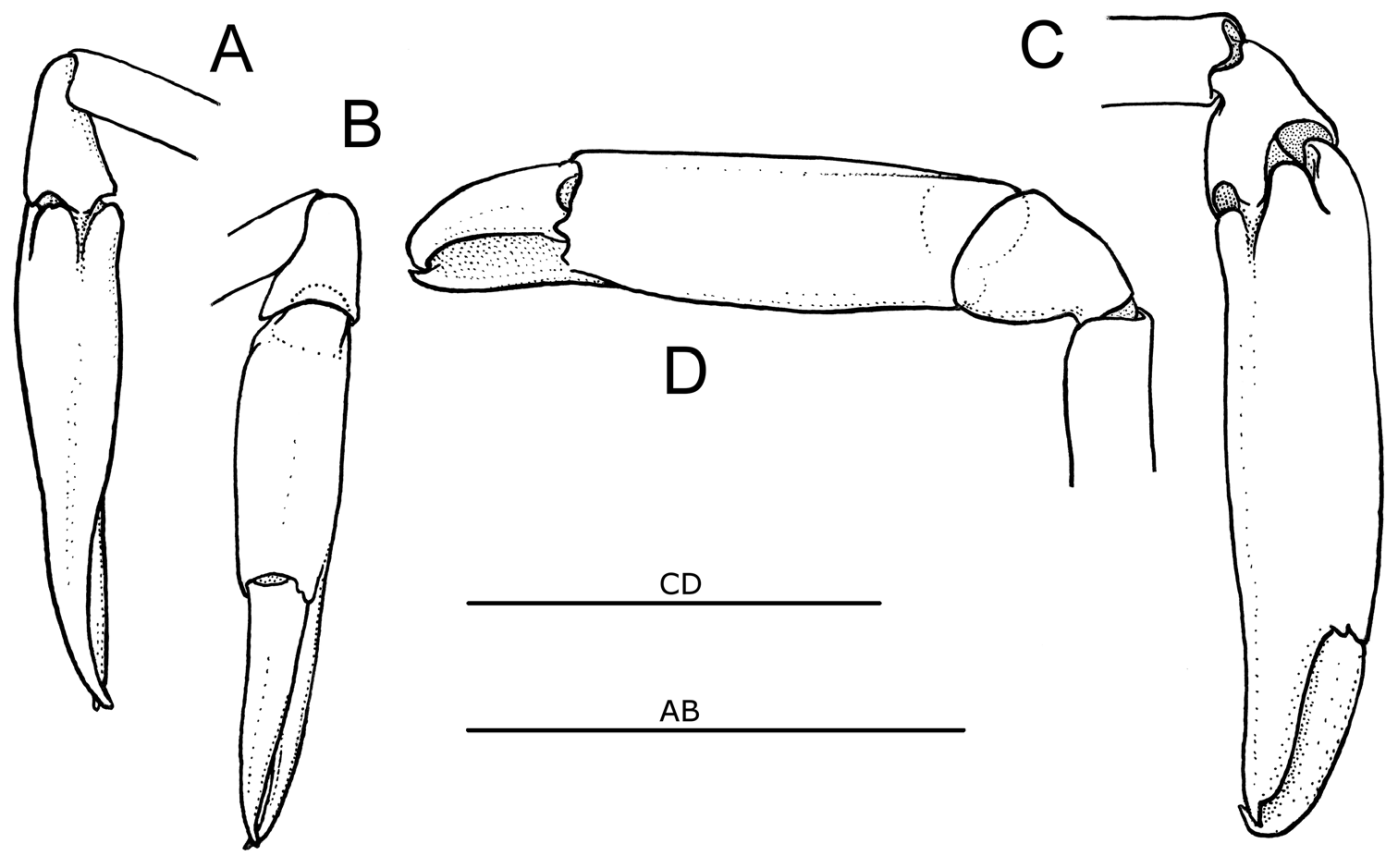
Second pereiopod of species of the genus *Cristimanus* gen. n. showing specific carpo-propodal articulation. **A, B** *Cristimenes commensalis* (Borradaile, 1915), comb. n., MTQ 33230, Lizard Island, Great Barrier Reef **C, D** *Cristimenes cristimanus* (Bruce, 1965), comb. n., UO 103-Vn08, Nhatrang Bay, Vietnam. (**A, C** medial aspect; **B, D** lateral aspect; scale bars 1 mm).

**Figure 3.**
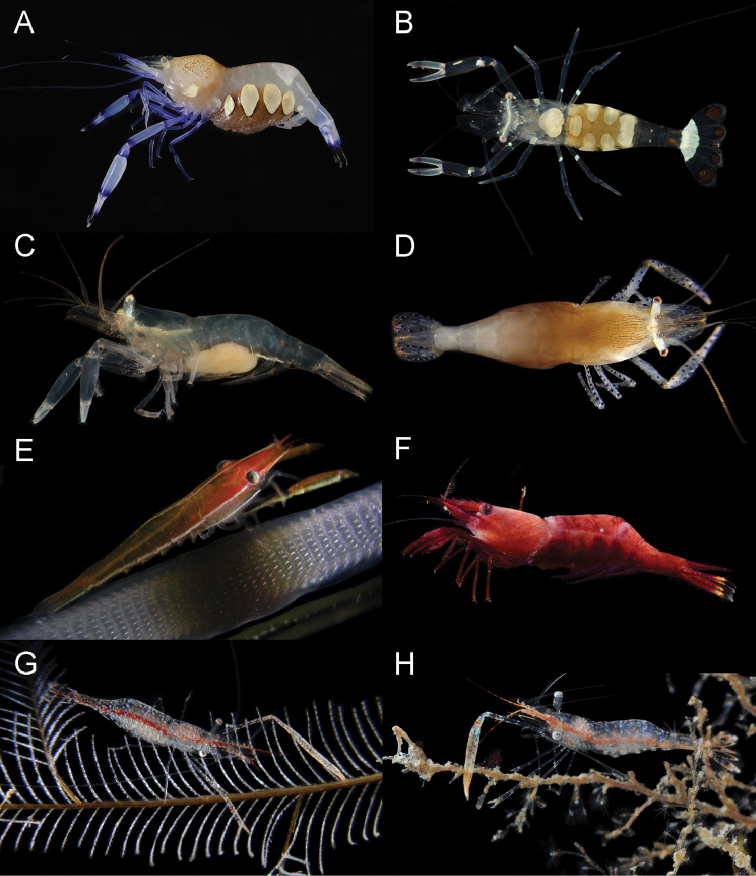
Examples of species from the genera reported in this study. **A, B** *Ancylocaris brevicarpalis* Schenkel, 1902: ovigerous females, MNHN-2014-558 and -156 (resp.), Kavieng, Papua New Guinea **C** *Actinimenes inornatus* (Kemp, 1922), comb. n., MNHN-IU-2014-315, with bopyrid parasite, Kavieng, Papua New Guinea **D** *Actinimenes ornatus* (Bruce, 1969), comb. n., ovigerous female, UO 80-Vn08, Nhatrang Bay, Vietnam **E** *Cristimenes cristimanus* (Bruce, 1965), comb. n., UO 103-Vn08, Nhatrang Bay, Vietnam **F** *Cristimenes commensalis* (Borradaile, 1915), comb. n., MTQ 33230, Lizard Island, Great Barrier Ree **G** *Rapimenes laevimanus* (Ďuriš, 2010), comb. n., ovigerous female holotype, RMNH D.53129, Nhatrang Bay, Vietnam **H** *Rapimenes granulimanus* (Bruce, 1978), comb. n., ovigerous female, MNHN-IU-2013-509, Madang, Papua New Guinea. (Photos: **A–G** Z Ďuriš; **H** A Anker).

*Cristimenes commensalis* (Borradaile, 1915), comb. n. (Figs 2A, B, 3F); *Cristimenes cristimanus* (Bruce, 1965), comb. n. (Figs 2C, D, 3E); and *Cristimenes zanzibaricus* (Bruce, 1967), comb. n.

##### Diagnosis.

Carapace smooth; rostrum well developed, subequal to antennular peduncle, compressed, usually with 5–7 low dorsal and 0–3 ventral teeth, lateral carinae with depressed supraorbital tooth, orbit feebly developed, epigastric tooth absent, inferior angle distinct, hepatic tooth close to antennal tooth and slightly lower positioned. Pleon smooth, fourth and ﬁfth pleura posteroventrally angulate. Telson with two pairs of small dorsal spines on posterior half, and with three pairs of posterior spines. Eyes with globular cornea. Basal antennular segment with 2–3 acute distolateral teeth. Antenna with basicerite unarmed, scaphocerite moderately broad, with distolateral tooth small, not reaching distal level of lamina. Epistome with pair of lateral rounded tubercles. Mandible without palp, molar process robust, incisor process normal, with 3–4 terminal teeth; maxillula with bilobed palp, laciniae moderately broad; maxilla with simple palp, basal endite slender, feebly bilobed or simple, coxal endite obsolete, scaphognathite moderately broad; ﬁrst maxilliped with simple palp, basal and coxal endites fused, exopod with distinct caridean lobe, ﬂagellum slender with 4 plumose distal setae, epipod bilobed; second maxilliped with normal endopod, propodus feebly produced medially, exopod similar to flagellum of ﬁrst maxilliped, coxa with elongate epipod without podobranch, arthrobranch rudimentary; third maxilliped with slender endopod, ischiomerus distinct from basis, exopod as in second maxilliped, coxa with large subcircular lateral plate, arthrobranch rudimentary. Fourth thoracic sternite without median process. First pereiopods slender, chela with ﬁngers tapering distally and feebly or distinctly subspatulate with entire cutting edges, coxa with obsolete distoventral lobe. Second pereiopods similar and subequal; cutting edges of ﬁngers dentate or denticulate; palm elongate, subequal or longer than fingers, subterminally articulated to short cup-shaped carpus, with pair of proximal lobes fitting dorsally to carpal cavity; carpus and merus unarmed. Ambulatory pereiopods slender, dactyli bi- or triunguiculate (i.e. with or without dorsal spinule behind unguis), unguis long, almost subequal to corpus length; propodus with ventral spinules and tufts of soft setae. Uropodal exopod elongate, laterally straight, with small distolateral tooth with mobile spine medially.

##### Etymology.

A combination of the subgeneric name *Cristiger* (see below) proposed by Borradaile, 1915 and *Periclimenes* in which genus the species were previously placed; gender masculine. As suggested by Holthuis (1993), the etymology of the name *Cristiger* (Latin = crest-bearer) was possibly in reference to the convex upper margin of the rostrum in the type species (from *crista* = crest, and *gero* = to bear).

##### Figures

**(selected**). Holthuis (1952: figs 18–19), Miyake and Fujino (1968: fig. 2e–g), Bruce (1965: figs 1–2; 1967: figs 26–29; 1982: fig. 2).

##### Systematic position.

The present new genus is closely related to three crinoid-associated genera, *Araiopontonia* Fujino & Miyake, 1970, *Laomenes* AH Clark, 1919, and *Unguicaris* Marin & Chan, 2006. This was already suggested by Marin and Chan (2006), and later supported by phylogenetic analyses (Kou et al. 2013, Gan et al. 2015, Horká et al. 2016). All these echinoderm-associated shrimps have a well-developed, somewhat downturned rostrum, generally dentate both dorsally and ventrally; the epistome is with a pair of lateral lobes (low, rounded in *Cristimenes* gen. n. and *Araiopontonia*, but acutely produced in the remaining genera); similar and subequal second pereiopods, and ambulatory legs possessing a specific type of ‘triunguiculate’ dactylus with a long main unguis. Such dactyli are secondarily reduced to a more biunguiculate state by the reduction of the dorsal spinule in the echinoid-associated species *Cristimenes cristimanus* and *Cristimenes zanzibaricus*, as well as in the crinoid associated genus *Laomenes*. The depressed supraorbital teeth associated with the lateral rostral carina are a synapomorphic character for the group, however, secondarily lost in *Unguicaris*. Such a reduction of the supraorbital teeth and lateral carinae was illustrated in some specimens of *Cristimenes commensalis* by Monod (1976), or Bruce (1982).

*Cristimenes* gen. n., together with *Araiopontonia*, can be distinguished from the genera *Laomenes* and *Unguicaris* by the rounded lateral lobes on the epistome (vs. acute projecting lobes). The new genus differs from all the three genera by a 3-dentate mandibular incisor (vs. distally expanded, multidentate), and by the unique carpo-propodal articulation of the second pereiopods, with the subterminal proximo-ventral articulation on the propodus leaving a distinctive posterior part of the propodus dorsally overhanging the articulation (Fig. 2). This lobe is deeply subdivided by a short but deep longitudinal groove into a pair of lobes which smoothly fit into the dorsal cavity of the short cup-shaped carpus when the propodus is extended anteriad. The proximal end of the propodus is then well hidden inside the carpus from dorsal view.

##### Range.

Widely distributed throughout the whole Indo-West Pacific region.

##### Ecology.

The genus *Cristimenes* comprises a single crinoid-associated species, *Cristimenes commensalis* comb. n., with the other two species, *Cristimenes cristimanus* comb. n., and *Cristimenes zanzibaricus* comb. n., living on echinoids (Echinodermata: Crinoidea, Echinoidea).

##### Remarks.

The genus *Cristimenes* is established here for three species, with Periclimenes (Cristiger) commensalis as the type species. This species was designated as the type species of the subgenus *Cristiger* Borradaile, 1915 by Holthuis (1955) since the previous designation of *Alpheus scriptus* Risso, 1822 by Borradaile (1917) as the type was invalid. As pointed out by Holthuis (1955, 1993), the name *Cristiger* Borradaile is a junior homonym of *Cristiger* Gistel, 1848 (Hymenoptera), and thus not available to be used for the present genus.

##### Key to species identification of *Cristimenes* gen. n.

**Table:** 

1	Ambulatory dactyli triunguiculate; associated with crinoids (basal antennular segment with 2–3 acute teeth distolaterally; first pereiopod fingers simple, subequal to palm; second pereiopods with cutting edges of ﬁngers dentate proximally and denticulate distally)	***Cristimenes commensalis* (Borradaile, 1915), comb. n.**
–	Ambulatory dactyli biunguiculate; associated with echinoids	**2**
2	Palm and dactylus of first pereiopod strongly compressed, palm tuberculate dorsally, dactylus carinate medially; second pereiopods with cutting edges of ﬁngers dentate throughout; basal antennular segment with 3 acute teeth distolaterally	***Cristimenes cristimanus* (Bruce, 1965), comb. n.**
–	Palm and dactylus of first pereiopod normal, smooth dorsally, uncarinate; second pereiopods with cutting edges of ﬁngers dentate proximally and denticulate distally; basal antennular segment with rounded lobe and 2 acute teeth distolaterally	***Cristimenes zanzibaricus* (Bruce, 1967), comb. n.**

#### Rapimenes gen. n.

Taxon classificationAnimaliaDecapodaPalaemonidae

http://zoobank.org/5157C46E-9EC0-4D4A-A34E-CBA986D443A9

##### Type species.

*Periclimenes granulimanus* Bruce, 1978, by present designation.

##### Included species.

*Rapimenes brucei* (Ďuriš, 1990), comb. n.; *Rapimenes granulimanus* (Bruce, 1978), comb. n. (Fig. 3H); and *Rapimenes laevimanus* (Ďuriš, 2010), comb. n. (Fig. 3G).

##### Material examined.

In addition to the type series (Ďuriš 2010), the following specimens were subsequently examined: ***Rapimenes granulimanus*** – 6 spms, (MNHN-IU-2013-10931), 1 spm. (IU-2013-11097), 2 spms (IU-2013-11077), *Papua Niugini Expedition* 2012, Madang Lagoon, Papua New Guinea, Dec. 2012, coll. Z Ďuriš. — ***Rapimenes laevimanus***– 3 spms (UO-Jp2012), Nago-city, Okinawa, Japan, 26°33,6'N, 127°57.6'E, 10 May 2012, depth 30 m, on sea pen cf. *Stylatula* sp. [Pennatulacea]; coll.: N Sirakawa & Y Yamada, lgt. R Minemizu.—1 spm. SW Taiwan, coll. C-W Lin (fcn 20130202-08).

##### Diagnosis.

Medium sized shrimps. Carapace smooth, with antennal and hepatic teeth; epigastric tooth lacking or, if present, clearly separated from them; hepatic tooth subequal and situated posteriorly of antennal tooth and slightly below. Rostrum slender, dorsal lamina bearing 6–10 teeth, ventral lamina obsolete, with 1–2 subterminal teeth. Inferior orbital angle produced, rounded. Pleon smooth, pleura posteroventrally rounded; telson slender, tapering distally, with 1–2 pairs of small dorsal spines and three pairs of posterior marginal spines. Ophthalmic somite without interocular process. Antennula and antenna normal, scaphocerite 3–4 times longer than broad. Eye with globular cornea and small accessory pigment spot, stalk distinctly longer than corneal diameter. Mandible without palp, incisor and molar processes stout. Maxillula with bilobed palp, upper and lower laciniae well developed. Maxilla with slender palp, well developed scaphognathite, distal (basal) endite bilobed; proximal (coxal) endite lacking. First maxilliped with simple palp, basal endite broad, coxal endite feebly demarcated, exopod well developed, caridean lobe normal, epipod distally bilobed. Second maxilliped with normal endopod and exopod, epipod small, simple, without podobranch. Third maxilliped with slender segments, ischiomerus and basis fused; exopod well developed, coxa with rounded lateral plate, single small arthrobranch present. Fourth thoracic sternites without special structures. First pereiopods slender, fingers narrow, simple, with dense tufts of long setae on sides, coxa with or without distoventral setose process, basis unarmed. Second pereiopods long and slender, distinctly unequal in length; major pereiopod overreaching scaphocerite by distal part of merus in adults; fingers simple, cutting edges entire or with 1–2 feebly developed teeth on proximal third of minor chela, and with 2–4 obtuse proximal teeth on major chela fingers; major pereiopod with palm 2.5–5 times longer than fingers. Ambulatory pereiopods slender, propodus with prehensile structure of long straight distoventral spines arranged to 2–5 pairs, spines longer than distal propodal depth; dactyli slender and curved, simple, or with distinct or minute distoventral tooth. Endopod of first male pleopod with angulate apex and distinct medial lobe; second male pleopod with appendix masculina with 3 terminal serrated setae and 2 lateral setae. Uropods normal; distolateral angle of exopod with small tooth and movable spine medially.

##### Etymology.

Combination of *rapina*, Latin for claw, to point on the prehensile structures on the ambulatory legs, and the name of the genus *Periclimenes* Costa, 1844, from which the new genus is separated; gender masculine.

##### Figures

**(selected**). Bruce (1978: figs 16–19), Ďuriš (1990: figs 1–2; 2010: figs 1–8).

##### Systematic position.

Based on the recent molecular phylogeny in Horká et al. (2016), the therein included two species of *Rapimenes* gen. n. are closely related to the genus *Phycomenes* Bruce, 2008, and to a pair of further *Periclimenes* spp., i.e. *Periclimenes affinis*, and *Periclimenes kallisto*, positioned near to *Ancylocaris brevicarpalis* comb. n. From *Phycomenes*, the species of the genus *Rapimenes* gen. n., can be easily distinguished by their larger size, distinctly unequal second pereiopods, prehensile ambulatory legs, and lack of a triangular process on the fourth thoracic sternum (see Bruce 2008b, Ďuriš 2010). Morphologically, the new genus (i.e. the previous “*Periclimenes granulimanus* group”) is phylogenetically close to the “*Periclimenes obscurus* group”, as already indicated by Eilbracht and Fransen (2015) who also listed the characters distinguishing all species of those two groups. Included in the “*Periclimenes obscurus* group” are some species which show distinct similarities to *Rapimenes*, such as *Periclimenes macrorhynchia* Eilbracht & Fransen, 2015, *Periclimenes nomadophila* Berggren, 1994, and *Periclimenes tonga* Bruce, 1988 (the latter synonymized with *Periclimenes granulimanus* by Marin 2012, but regarded as valid by Eilbracht and Fransen 2015), particularly in the shape and proportions of the pereiopods and rostrum. These species have biunguiculate walking dactyli, sternal thoracic ridges, and distinct setose coxal lobe on the first pereiopod, i.e. the characters typical for the “*Periclimenes obscurus* group”. The ambulatory dactyli vary in *Rapimenes* spp. from simple to bearing a minute additional tooth; the latter is true for *Rapimenes brucei*, new comb., for which a coxal setose lobe also was reported (Ďuriš 1990). It is thus possible that both species groups represent a common evolutionary clade which inner diversity is still to be resolved.

##### Distribution.

Madagascar; Maldive Islands; Indonesia; Vietnam and Taiwan, South China Sea; Japan; Heron Island, Great Barrier Reef, Australia.

##### Ecology.

The species of the present genus have been recorded as associated with antipatharians, hydroids, pennatularians, and scyphozoans (Cnidaria) (Bruce 1978, 1988, Ďuriš 1990, 2010, Minemizu 2013, Eilbracht and Fransen 2015).

##### Remarks.

The generic name *Rapimenes* was used as a *nomen nudum* by Marin (2009: 15) in reference to the then undescribed species *Periclimenes laevimanus* Ďuriš (2010), as “*Rapimenes laevimanus* Ďuriš & Petrusek (in press)”. The name is herein validly reinstated for the genus, indeed now containing *Rapimenes laevimanus*.

##### Key to species identification of *Rapimenes* gen. n.

(modified from Ďuriš 2010).

**Table:** 

1	Major second pereiopod extremely long and slender, overreaching scaphocerite by proximal merus, carpus longer than both chela or merus; walking dactyli feebly biunguiculate with small distoventral tooth on corpus, propodi with long spines arranged to 4 distoventral pairs	***Rapimenes brucei* (Ďuriš, 1990), comb. n.**
–	Second pereiopods unequal, slender, at least overreaching scaphocerite by distal merus, carpus subequal or distinctly shorter than both chela or merus; walking dactyli simple or with rudimentary distoventral tooth, propodi with 1–3 single proximal spines in addition to 2–4 distoventral pairs of long spines	**2**
2	Major second pereiopod with palm granulate; minor second chela with 1–2 teeth on cutting edges	***Rapimenes granulimanus* (Bruce, 1978), comb. n.**
–	Major second pereiopod with palm smooth; minor second chela with cutting edges entir	***Rapimenes laevimanus* (Ďuriš, 2010), comb. n.**

## Supplementary Material

XML Treatment for Ancylocaris

XML Treatment for Actinimenes

XML Treatment for Cristimenes

XML Treatment for Rapimenes
